# The Importance of Oral Health in Immigrant and Refugee Children

**DOI:** 10.3390/children6090102

**Published:** 2019-09-09

**Authors:** Eileen Crespo

**Affiliations:** Department of Pediatrics, Hennepin County Medical Center, Minneapolis, MN 55415, USA; eileen.crespo@hcmed.org

**Keywords:** oral health, immigrant and refugee children, culturally responsive care, acculturation

## Abstract

According to the Migration Policy Institute, 2017 data revealed that a historic high 44.5 million people living in the United States (US) were foreign-born (Zong, J., et.al., 2019), more than double the number from 1990 (U.S. Immigrant Population and Share over Time. 1850-Present, 2019). Since the creation of the Refugee Resettlement Program in 1980, refugee families have settled in the US more than in any other country in the world (Radford, J., 2019). In 2018, for the first time, Canada overtook the US in numbers of refugees accepted (Zong, J., et. al., 2019). Foreign-born people now account for 13.7% of the total US population (Zong, J., et. al., 2019). Further, a quarter of children in the United States currently live in households with at least one foreign-born parent (America’s Children in Brief: Key National Indicators of Well-Being, 2018). These population shifts are important to note because immigrant and refugee families bring cultural influences and health experiences from their home countries which can greatly affect the overall health and well-being of children. For these new arrivals, oral health is often a significant health issue. The severity of dental disease varies with country of origin as well as cultural beliefs that can hinder access to care even once it is available to them (Obeng, C.S. Culture and dental health among African immigrant school-aged children in the United States, 200; Tiwari, T.; Albino, J. Acculturation and Pediatric Minority Oral Health Interventions, 2017). As pediatricians and primary care providers, we should acknowledge that oral health is important and impacts overall health. Healthcare providers should be able to recognize oral health problems, make appropriate referrals, and effectively communicate with families to address knowledge gaps in high-risk communities.

## 1. Background

Latinos are the largest ethnic group in the US and are projected to be the dominant minority group by 2044 [1]. Mexico accounts for the largest group of immigrants in the US, responsible for 25% of all immigrants [2]. However, there has been a shift noted since 2010 with larger numbers of immigrants coming from countries in South and East Asia such as India and China [2]. The number of immigrants of Asian origin is growing so quickly that they are expected to surpass Latinos to become the largest immigrant group in the United States by 2055 [2]. Additionally, because foreign-born women tend to have higher fertility rates [3], it is projected that by 2020, more than half of the children in the United States will have parents who are foreign-born [4].

According to 2015–2016 data from the National Health and Nutrition Examination Survey (NHANES), Hispanic children have the highest prevalence of dental caries of children aged 2–19 years in the US [5]. The rates for Hispanic children were higher than for all other groups surveyed at 57.1% and significantly higher than for non-Hispanic white children [5]. It Is important to note that as the largest immigrant group in the US that also suffers from the highest prevalence of dental disease, children of Hispanic immigrants may benefit from enhanced oral health screening and appropriate referral in the primary care setting.

Refugees coming to the US are another group at high risk of caries. A recent study showed a 64% higher caries risk rates in refugee children from Asia compared with African refugee children [6]. The refugee children from Asia also had more urgent unmet dental needs than refugees from Africa, 46.1% vs. 30%, respectively [6]. According to 2018 Pew Research data, the majority of refugees to the US are arriving from Africa, Eastern Europe, the Middle East, and Asia [4]. Since the United States government started the Refugee Resettlement Program, three million refugees have come to the US. While the total number of refugees is lower than that of immigrants, refugees can be at higher risk for poor oral health due to multiple factors. Some of these factors, which are described in detail, include home country, cultural influences including diet and health beliefs, as well as time spent in refugee camps where cariogenic foods have historically been available [7].

## 2. Role of Country of Origin

Country of origin has an impact on the rate of dental caries because access to dental care, traditional diet, access to refined sugars, exposure to fluoride, and personal hygiene practices all affect the risk of developing caries. Cote and colleagues found marked differences in groups of refugee children screened for dental caries upon arrival in the US. In the population studied, refugee children from East African countries had surprisingly low rates of caries, even lower than African-American children from the US, despite many of them having never had a dental visit nor practiced routine dental hygiene [8]. Refugee children from Eastern European countries had the highest rates of caries experience in this study. Refugee children from Eastern European countries were 5.6 times more likely to have caries when compared to African refugees, and they were 4.7 times more likely to have unmet dental needs despite having more access to dentists in their home countries [8]. Ogawa and colleagues noted the same protective factors and low rates of caries for refugees from Africa more than ten years later [6]. These variations among groups highlight the importance of understanding the role of early health experiences and risk factors for dental caries which can be geographically associated.

Other factors can contribute to specific oral health patterns in certain refugee and immigrant groups. Children from East African countries, in particular, have been found to be naturally exposed to optimal or even high levels of fluoride in water supplies [6,8]. Further, East African children from Somalia and Ethiopia are raised on a traditional diet that is very low in sugar and have cultural practices, such as the use of chewing sticks, that tend to spare them from high rates of caries [6,7,8]. Children from Eastern European countries have the opposite experience with low rates of fluoride found in water sources and high rates of dental disease [8]. There are also variations within countries that may increase caries risk dependent on family socioeconomic status with children from urban areas and more affluent families exhibiting higher rates of caries because of increased access to sweets and sugary beverages [7,8]. Knowledge of regional variations in the incidence of caries and other cultural factors that impact oral health can help to tailor health messaging to be more effective.

## 3. Role of Acculturation

Arrival in the United States is a time of stress and adjustment for refugee and immigrant families. Acculturation, defined as “lifestyle and behavioral changes of people as they move from one culture and adapt to another culture, usually as a result of immigration,” [9] affects oral health. One of the most significant differences for newly arriving groups in regard to attitudes about oral health is a culture shift to an emphasis on healthy primary teeth. Refugee and immigrant families often arrive from home countries that do not emphasize oral health or even value healthy primary teeth [10,11,12,13]. A review of studies on acculturation and the impact on oral health found that in general, increasing acculturation results in improved oral health [9]. Using proxy measures of length of residence and English proficiency reveals improved adherence with preventive dental care for Latino and Chinese immigrant families the longer they reside in the US.

An important aspect that may contribute to slower adoption of health messaging is the common belief of fatalism in certain health outcomes, especially dental caries [11,12,13,14]. Many cultures see dental caries as a rite of childhood and there may be no expectation of healthy primary teeth. This knowledge gap affects their belief in the value of preventive dental visits and may predispose already vulnerable populations to increased caries in primary and permanent teeth. Higher-risk communities may benefit from oral health messaging that is tailored within their cultural context and, ideally, presented from members of their own community to build trust and acceptance [14].

Living arrangements for many new arrivals can contribute to poor oral health. Many cultures rely on multigenerational housing with extended family such as grandparents, aunts, uncles and even older siblings providing childcare. Family members who care for young children influence daily routines and food choices, especially bottle use and sweet treats [12]. Many cultures also place great emphasis on respect for older members of their community [13]. Cultural clashes can be seen as families struggle with incorporating health norms from the US that may be at odds with the family elder’s belief or opinion. Thus, a grandparent who does not believe in the utility of preventive dental care can be a barrier to accessing care.

In many countries, preventive dental care is not a part of routine healthcare, and this may impact attitudes about when to seek care. One study suggests that Chinese immigrants may first look to homeopathic treatments and consider a dental visit only as a last resort [13]. The common belief that dental care is not necessary if a child is not complaining of pain has been noted in Chinese, Filipino, and African immigrant families [10,11,13], as well as Latinos [8]. One study of West African immigrants found the expense of dental care or insurance to conflict with urgent financial responsibilities related to family members in their home country [10]. Responses to a survey with the same group of West Africans revealed that 10% perceived dental care to be unimportant, with 70% reporting that dental decay was not as urgent a disease as others, such as HIV [10]. For some who did access care, caregivers reported they did so to avoid appearing negligent with local social service agencies [10]. These specific cultural beliefs require detailed conversations and information for families to understand the focus on prevention. Additionally, many refugee and immigrant families qualify for public health insurance programs that provide dental care at low or no cost.

However, a recent US federal government policy change known as the public charge rule may significantly impact utilization of public health insurance programs and, therefore, access to dental services. This policy expands the types of government-funded benefits used to determine whether an immigrant is likely to become primarily dependent on public funding [15]. In particular, health insurance, along with food and housing assistance, will be included in the policy expansion. Though the rule change does not apply to refugees or legal immigrant children under age 21, many families may be confused or fearful that the use of these services may negatively impact their permanent residency application [15,16]. While the rule change is currently being contested in multiple states, barring successful legal rulings, the change will become effective 15 October 2019. By some reports, even the threat of the rule change has already resulted in decreased enrollment in public assistance programs [16].

## 4. Role of Diet

While multiple factors contribute to the pathophysiology of caries formation, one of the principal causes is dietary sugar intake [7]. Amount and frequency of sugar consumption along with host and environmental factors contribute to caries development. Knowledge gaps regarding risks as well as easy access to processed sugary foods and beverages in the United States result in high sugar diets in immigrant and refugee families. Studies show there is a lack of awareness regarding dietary habits—including unrestricted snacking—that contribute to caries [14,17]. In one study, Mexican-American mothers identified candy and sugary beverages as cariogenic but did not always differentiate the role of carbohydrates in crackers, breads, and cookies [18]. Many newcomer families can benefit from specific information about all sources of excess carbohydrates as they may substitute salty crackers or sweetened yogurt drinks, intending to give their child healthier food options.

Prolonged bottle use is common in certain cultures and is extremely prevalent in Latino and Asian families. Beginning in infancy, immigrant and refugee groups may perceive that infant formula is more convenient [11] or more nutritious [12]. These health beliefs may cause families to choose to feed their children formula over breastfeeding and to bottle-feed for longer periods than recommended. One study showed that 36.8% of Mexican-American children were still drinking from a bottle at 24–48 months of age [19]. This rate was more than double the rate of White and African-American children [19]. In fact, in Latino households, it is typical to calm a crying toddler with bottle of sweetened milk or juice [12,18]. As mentioned previously, since many immigrant households tend to be multifamily or multigenerational, it may be a priority to manage a crying child who keeps others awake. Consequently, giving specific messages about the risks of prolonged bottle feeding with sugary liquids including cow’s milk and juice, as well as discussing behavior strategies for trained night feeders, is more practical than simply recommending that they stop bottle feeding. Further, it is important to address that shifting to sippy cups if still filled with sugary liquids will not eliminate the risks of caries, as many mothers may assume [18].

## 5. Role of Culturally Responsive Care

A detailed review of the need for provider diversity as well as patient preference for a culturally-concordant provider is beyond the scope of this article. However, the concept of culturally responsive care has been recognized as integral to effective partnerships with families and can significantly impact care delivery [14]. Culturally responsive care is healthcare that accepts that the patient’s home culture is important and affects the way individuals interact with health systems [20]. An additional barrier that may shape dental care access is that immigrant and refugee groups may lack trust in dental providers, and many endorse fear from dental experiences in their home country, which may contribute to lower use of preventive dental services [13]. Working with diverse populations requires insight into cultural beliefs and practices [13], as well as history of health experiences. Acknowledging that providers have their own culture and biases that may affect patient encounters is another important aspect of culturally responsive care.

Though taking into account all of the nuances of culturally responsive care is important, lack of English proficiency remains a significant barrier to healthcare access [21]. Patients with language barriers have difficulty accessing services and are vulnerable to higher rates of adverse health outcomes [21]. Utilizing the 2007 National Survey of Children’s health, Avila and Bramlett found that the largest health disparities were seen for Hispanic children who were recent arrivals and those children living in non-English speaking households [22]. This is critically important since, according to 2017 Pew Research, Mexican immigrants in the US have the lowest rates of English proficiency of any ethnic group [3]. Immigrants from Europe, Canada, Sub-Saharan Africa, and the Middle East have the highest rates of English proficiency [3]. Addressing language barriers with trained medical interpreters is a best practice that is legally mandated in hospitals that receive federal funding [21]. Professional language interpretation should be made available in all healthcare settings.

Dental education has incorporated culturally responsive care as a requirement for training [23]. Dental education is shifting to a multidisciplinary approach that emphasizes the humanistic side of patient care as the foundation of understanding. Overlaying the complex interplay of the patient’s individual culture combined with societal influence and acculturation provides a more complete appreciation of the patient and their view of oral health. In their paper, Donate-Bartfield and colleagues emphasized the importance of presenting education on behavioral and ethical concepts that value patient autonomy over historical paternalism [23]. These educational concepts are more effective when introduced prior to community-based learning experiences where dental students care for culturally-diverse patients. Service-learning assignments are an excellent way for students to practice compassionate care while still in an educational setting [14].

Medical schools and health systems have also tried to address cultural insensitivity as well as the racial bias and stereotyping that has impacted health equity for many years [24,25,26,27]. Efforts have included medical school and residency curriculum on the care of minority populations and the effects of health disparities [25]. The Office of Minority Health has developed standards for healthcare delivery that is culturally and linguistically sensitive [24]. The standards include a variety of initiatives including a requirement that oral and written notices be printed in multiple languages, developing recruitment strategies for diverse professional as well as support staff, regular educational programming for clinic and hospital personnel, as well as procedures to address cross-cultural conflicts [24]. These standards represent a framework to help build an awareness of the influence of culture on behaviors and access to healthcare.

## 6. Conclusions

Multiple factors influence the development of dental caries and oral health problems in immigrant and refugee children. Knowledge of protective factors as well as the important role culture plays in the way different ethnic communities interact with health systems can help improve outcomes for high risk populations. Healthcare providers in all disciplines should strive to build therapeutic relationships with at-risk populations and deliver care that is culturally appropriate and can address oral health disparities.

To improve the oral health of immigrant and refugee children, medical providers should:

(1) Ask about diet, specifically about exposure to cariogenic foods and drinks, bottle use, and history of dental care;
(2) Examine the teeth of immigrant and refugee children assessing for white spot lesions and frank decay;
(3) Apply fluoride varnish during primary care visits;
(4) Counsel families on the importance of daily oral hygiene practices, use and amount of fluoridated toothpaste, and encourage fluoridated tap water;
(5) Refer with appropriate urgency to dental providers in your community.

## References

[1] Colby S.L., Ortman J.M. (2014). Projections of the Size and Composition of the U.S. Population: 2014 to 2060.

[2] Zong J., Batalova J., Burrows M. (2019). Frequently Requested Statistics on Immigrants and Immigration in the United States. https://www.migrationpolicy.org/article/frequently-requested-statistics-immigrants-and-immigration-united-states#Now.

[3] Radford J. (2019). Key Findings about U.S. Immigrants. Pew Research Center. https://www.pewresearch.org/fact-tank/2019/06/03/key-findings-about-u-s-immigrants/.

[4] Older People Projected to Outnumber Children for First Time in U.S. History 13 March 2018. https://www.census.gov/newsroom/press-releases/2018/cb18-41-population-projections.html.

[5] Fleming E., Afful J. (2018). Prevalence of Total and Untreated Dental Caries among Youth: United States, 2015–2016.

[6] Ogawa J.T., Kiang J., Watts D.J., Hirway P., Lewis C. (2019). Oral Health and Dental Clinic Attendance in Pediatric Refugees. Pediatr. Dent..

[7] Holm A.K. (1990). Diet and Caries in High-Risk Groups in Developed and Developing Countries. Caries Res..

[8] Cote S., Geltman P., Nunn M., Lituri K., Henshaw M., Garcia R.I. (2004). Dental Caries of Refugee Children Compared with US Children. Pediatrics.

[9] Gao X.L., McGrath C. (2011). A Review of the Oral Health Impacts of Acculturation. J. Immigr. Minor. Health.

[10] Obeng C.S. (2007). Culture and dental health among African immigrant school-aged children in the United States. Health Educ..

[11] Tiwari T., Albino J. (2017). Acculturation and Pediatric Minority Oral Health Interventions. Dent. Clin..

[12] Ng M.W. (2003). Multicultural influences on child-rearing practices: Implications for today’s pediatric dentist. Pediatr. Dent..

[13] Hilton I.V., Stephen S., Barker J.C., Weintraub J.A. (2007). Cultural factors and children’s oral health care: A qualitative study of carers of young children. Community Dent. Oral Epidemiol..

[14] Garcia R.I., Cadoret C.A., Henshaw M. (2008). Multicultural Issues in Oral Health. Dent. Clin. N. Am..

[15] Immigrant Legal Resource Center Public Charge. https://www.ilrc.org/public-charge.

[16] Dewey C. (2017). Immigrants are Going Hungry so Trump Won’t Deport Them. The Washington Post.

[17] Chomitz V.R., Park H.J., Koch-Weser S., Chui K.K.H., Sun L., Malone M.E., Palmer C., Loo C.Y., Must A. (2019). Modifying dietary risk behaviors to prevent obesity and dental caries in very young children: Results of the Baby Steps to Health pediatric dental pilot. J. Public Health Dent..

[18] Hoeft K.S., Barker J.C., Masterson E.E. (2010). Urban Mexican-American mothers’ belief about caries etiology in children. Community Dent. Oral Epidemiol..

[19] Brotanek J.M., Halterman J.S., Auinger P., Flores G., Weitzman M. (2005). Iron deficiency, prolonged bottle-feeding and racial-ethnic disparities in young children. Arch. Pediatr. Adolesc. Med..

[20] Carteret M. (2008). Cross-Cultural Values of Latino Families. Dimensions of Culture. https://www.dimensionsofculture.com/2010/10/576/.

[21] Goenka P. (2016). Lost in translation: Impact of language barriers on children’s healthcare. Curr. Opin. Pediatr..

[22] Avila R.M., Bramlett M.D. (2013). Language and Immigrant Status Effects on Disparities in Hispanic Children’s Health Status and Access to Health Care. Matern. Child Health J..

[23] Donate-Bartfield E., Lobb W.K., Roucka T.M. (2014). Teaching Culturally Sensitive Care to Dental Students: A Multidisciplinary Approach. J. Dent. Educ..

[24] Chin J.L. (2000). Culturally competent health care. Public Health Rep..

[25] Van Ryn M., Burke J. (2000). The effect of patient race and socioeconomic status on physicians’ perceptions of patients. Soc. Sci. Med..

[26] Peña Dolhun E., Muñoz C., Grumbach K. (2003). Cross-Cultural Education in U.S. Medical Schools: Development of an Assessment Tool. Acad. Med..

[27] Geiger H.J., Borchelt G. (2003). Racial and ethnic disparities in US health care. Lancet.

